# Identification and prenatal diagnosis of a novel likely pathogenic ANOS1 variant in a large Chinese Kallmann syndrome family

**DOI:** 10.3389/fgene.2026.1870344

**Published:** 2026-08-05

**Authors:** Xiulan Hao, Yanchou Ye, Mancheng Liu, Zhechao Zhang, Haofeng Ning, Xiaonan Wang, Fangchao Tao, Xiujing Huang, Jingran Du, Tian Li

**Affiliations:** 1 Department of Obstetrics and Gynecology, The Seventh Affiliated Hospital, Sun Yat-sen University, Shenzhen, China; 2 Department of Obstetrics and Gynecology, Prenatal Diagnostic Center, The Seventh Affiliated Hospital, Sun Yat-sen University, Shenzhen, China

**Keywords:** ANOS1, co-segregation analysis, Kallmann syndrome, prenatal diagnosis, whole-genome sequencing

## Abstract

**Background:**

This study aimed to systematically analyze a Chinese family with Kallmann syndrome (KS), evaluate the pathogenicity of a candidate variant through co-segregation analysis and bioinformatic predictions, and provide clinical intervention for the family.

**Methods:**

This study included a Chinese family with KS comprising 40 members in total (37 living, 3 deceased). Whole-genome sequencing (WGS) was performed on the proband, followed by Sanger sequencing for co-segregation analysis with 22 family members. Multiple bioinformatics tools were used to assess evolutionary conservation, and AlphaFold3 modeling was employed to predict the potential impact on protein structure. Based on the genetic findings, prenatal diagnosis was subsequently offered to and performed for a female carrier in the family.

**Results:**

A hemizygous ANOS1:c.1879T>C (p.Ser627Pro) variant was identified. Among the eight males with clinical features of KS in this family, five of whom underwent genetic testing, and all carried the variant. The variant fully co-segregated with KS among the genotyped individuals: all five tested males carried the variant (with their mothers confirmed as carriers), while five unaffected male relatives were wild-type. Bioinformatics analysis predicted that the variant site is evolutionarily conserved across species. AlphaFold3 modeling further suggested that the p. Ser627Pro substitution disrupts the original hydrogen bonding network, potentially leading to protein instability and function impairment. Based on these genetic findings, prenatal diagnosis was performed for a female carrier in the family, and the results showed that the fetus did not carry the variant.

**Conclusion:**

Combining family co-segregation (PP1) and population frequency data (PM2), the ANOS1 c.1879T>C variant was classified as likely pathogenic, providing important information for genetic counseling. This study demonstrated that even in the absence of functional experiments, comprehensive family analysis can provide crucial clues for variant of uncertain significance (VUS) interpretation. This study also guided prenatal diagnosis, offering a referable strategy for the management of similar rare diseases.

## 1 Introduction

Congenital hypogonadotropic hypogonadism (CHH) is a paradigm of a rare disease in which understanding the genetic basis has profound implications for diagnosis, clinical management, and family planning. Characterized by incomplete or absent puberty and infertility, CHH results from a failure of the hypothalamic-pituitary-gonadal axis (Raivio and Miettinen, 2019). The classic clinical features of CHH include delayed or absent puberty, low levels of sex hormones and gonadotropins, and frequently associated conditions such as olfactory dysfunction and renal developmental anomalies (Stamou and Georgopoulos, 2018). According to the presence of olfactory defects, CHH can be further categorized into KS, which is associated with anosmia or hyposmia, and normosmic CHH (nCHH) (Wang et al., 2022).

ANOS1 was the first identified causative gene for X-linked recessive (XLR) CHH (Franco et al., 1991). The gene encodes anosmin-1, an extracellular matrix protein that plays a critical role during embryonic development. Anosmin-1 is widely expressed in multiple tissues including the olfactory bulb, cerebellum, spinal cord, inner ear, and kidneys, where it regulates neural cell adhesion, axonal guidance, and branching morphogenesis (Cho et al., 2019; Duittoz et al., 2022). This broad expression pattern explains the multisystem clinical manifestations frequently observed in ANOS1-related KS. Beyond hypogonadism and olfactory dysfunction, patients may present with mirror movements, hearing loss, and renal malformations such as unilateral renal agenesis.

ANOS1 exhibits a very high probability of loss of function (LOF) in tolerance (pLI), with a score approaching 1.0. This indicates that the gene is under strong selective pressure against mutations that disrupt protein function (Karczewski et al., 2020). Consistent with this, among the currently known ANOS1 variants cataloged in public databases such as ClinVar and the Human Gene Mutation Database (HGMD), the majority are established pathogenic LOF mutations, including nonsense, frameshift, and large deletion variants (Landrum et al., 2025; Stenson et al., 2020). However, despite the growing number of ANOS1 variants identified through next generation sequencing, a significant proportion remain insufficiently characterized. This is particularly true for missense variants, as well as those classified as VUS. Truncating mutations clearly ablate protein function. In contrast, missense variants may have unpredictable effects on protein structure, stability, or interactions with binding partners. As a result, their role in CHH pathogenesis still lacks systematic evaluation. Furthermore, the genotype-phenotype correlations for these variants remain largely unclear. This poses challenges for genetic counseling and clinical decision-making.

Therefore, this study analyzes a large Chinese KS family carrying an ANOS1 missense variant. By integrating systematic clinical phenotyping and familial co-segregation analysis, we aim to evaluate the pathogenicity of this variant. This study will help clarify the role of such variants in the pathogenesis of KS and provide precise genetic diagnosis and reproductive intervention options for the family.

## 2 Materials and methods

### 2.1 Subjects

This study investigated a family with a history of inherited genitourinary and olfactory abnormalities. The family was identified through a pregnant woman who underwent routine prenatal screening at the Prenatal Diagnostic Center of the Seventh Affiliated Hospital of Sun Yat-sen University. The familial pedigree spans four generations and includes a total of 40 family members (37 living, 3 deceased). Based on clinical diagnosis, there are eight affected males in this family: the pregnant woman’s two maternal uncles, her elder brother (proband), and five nephews (sons of her sisters). However, blood samples were available for only five of these eight affected males: the 49-year-old proband and four of the five nephews (aged 14, 16, 13, and 12 years; mean age of the four nephews 13.78 years). The two uncles and one nephew declined blood collection. All five sampled affected males carried the ANOS1:c.1879T>C variant, while five unaffected male relatives were wild-type. The mothers of the affected males were confirmed as carriers, demonstrating complete co-segregation with KS phenotype among the genotyped individuals. All affected male relatives exhibited micropenis and small testes. The clinical phenotypes are summarized in Table 1.

**TABLE 1:** Clinical profiles of five individuals with KS who underwent genetic sequencing.

No	Family ID	Age	Sex	Cryptorchidism	Micropenis and small testes	Absence of secondary sexual features (facial andaxillary hair growth and high-pitched voice)	Unilateral kidney	Synkinesia	Anosmia	Azoospermia
1	III2	49	M	Y	Y	Y	N	N	Y	Y
2	IV3	14	M	Y	Y	Y	N	Y	Y	—
3	IV5	16	M	Y	Y	Y	Absence of right kidney	Y	Y	—
4	IV7	13	M	Y	Y	Y	Absence of left kidney	Y	Y	—
5	IV14	12	M	Y	Y	Y	Absence of right kidney	N	Y	—

Abbreviations: M, male; Y, yes (presence of symptom/feature); N, no (absence of symptom/feature); /, not applicable or data not available (azoospermia not assessed in prepubertal males).

**FIGURE 1:**
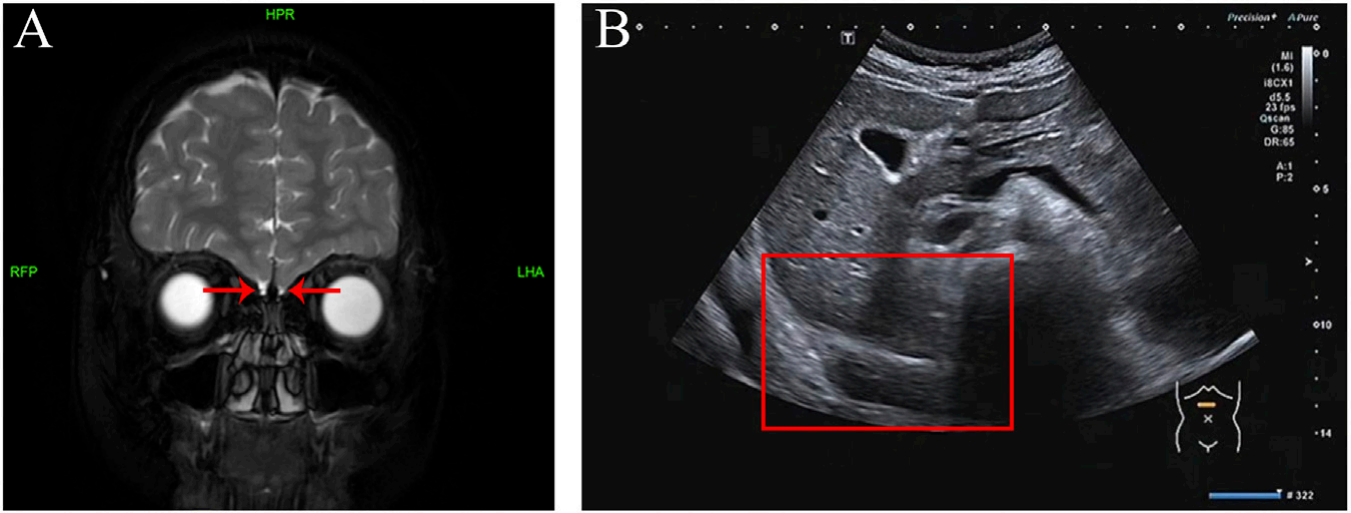
MRI and ultrasound images of the affected male patient (IV14). **(A)** Coronal thin-section T2-weighted images demonstrated no distinct nerve signal within the olfactory grooves bilaterally (indicated by red arrows). **(B)** Abdominal ultrasonography revealed absence of the right kidney in the right renal fossa, suggestive of right renal agenesis (indicated by red box).

One of the affected nephews (IV14, aged 12 years) underwent additional clinical evaluations, including brain MRI, B-ultrasound, and laboratory testing. Coronal thin-section T2-weighted images demonstrated no distinct nerve signal within the olfactory grooves bilaterally (Figure 1A). The patient also underwent B-ultrasound, which revealed right renal agenesis (Figure 1B). Laboratory testing of the individual was consistent with hypogonadotropic hypogonadism, with markedly low luteinizing hormone (LH, <0.09 mIU/mL) and follicle-stimulating hormone (FSH, 0.41 mIU/mL).

All participants provided written informed consent prior to enrollment. The study protocol was approved by the Medical Ethics Committee of the Seventh Affiliated Hospital, Sun Yat-sen University (certificate No: KY-2026-094-01).

### 2.2 Methods

#### 2.2.1 WGS

Whole-genome sequencing was performed on genomic DNA extracted from the proband’s peripheral blood. A total of 300 ng of gDNA was enzymatically fragmented, and fragments of 200–300 bp were selected using magnetic bead–based size selection. Following end repair and adapter ligation, the library was purified and polymerase chain reaction (PCR) amplified. Quality-controlled libraries were circularized and digested to generate DNA nanoballs (DNBs). The DNBs were pooled and sequenced on the DNBSEQ-T7 platform (PE100 + 10). All sequencing and bioinformatics analyses were conducted by BGI (Shenzhen, China). Sequencing quality control metrics: mean target region depth ≥40 X, with ≥90% of bases achieving a depth of ≥20 X. Raw reads were processed with SOAPnuke (Chen et al., 2018) and aligned to GRCh37/hg19 reference genome using BWA. Variant calling for SNVs and indels was performed with GATK, followed by annotation and filtering against population and disease databases. Variant interpretation was conducted according to the American College of Medical Genetics and Genomics (ACMG) guidelines (Richards et al., 2015). CNVkit was used to analyze and exclude copy number variant (CNV) from the WGS data.

#### 2.2.2 Sanger sequencing validation and family co-segregation analysis

To validate the variant identified by WGS and to assess its familial co-segregation with the phenotype, specific primers flanking the target genomic region of the ANOS1 variant were designed. Primer pairs (forward: 5′- CCGGCGTCCGGAACG-3′; reverse: 5′- GCT​GCC​AAG​AAT​TGA​GTG​AGG​TG-3′) were designed using Primer3 software based on the reference genome (GRCh37/hg19). PCR amplification was subsequently performed using genomic DNA extracted from peripheral blood lymphocytes of all 22 available family members. DNA extraction was carried out using the QIAamp DNA Blood Mini Kit (Qiagen). The thermal cycling conditions included an initial denaturation at 95 °C for 5 min, followed by 35 cycles of denaturation at 95 °C for 30 s, annealing at 60 °C for 30 s, and extension at 72 °C for 45 s, with a final extension at 72 °C for 10 min. The resulting amplicons were purified and subjected to Sanger sequencing on an ABI 3500 Dx DNA Analyzer. The obtained sequences were aligned to the reference sequence of ANOS1 (NM_000216.4) to confirm the findings from WGS.

#### 2.2.3 3D modeling of protein structure

To elucidate the structural impact of the identified variant on protein function and to assess its evolutionary conservation, we employed a combination of computational protein modeling and phylogenetic sequence analysis.

Computational modeling of the protein complex was performed with AlphaFold3 (Abramson et al., 2024). The model was visualized and rendered in PyMOL 2.5.3, with intermolecular interactions analyzed and highlighted. To assess the phylogenetic conservation of the affected amino acid residue, we performed a multi-sequence alignment. Homologous protein sequences from representative vertebrate species were retrieved from the UniProt (UniProt, 2025). The sequences were aligned using Clustal Omega (Li et al., 2015) with default parameters. The resulting alignment was visualized to examine the degree of conservation across species, with particular focus on the residue corresponding to the mutation site. All figures, including structural representations and sequence alignments, were annotated using Adobe illustrator.

#### 2.2.4 Prenatal diagnosis

To perform prenatal diagnosis for the fetus (pedigree member IV16), amniotic fluid cells were obtained from the mother (pedigree member III8) by transabdominal amniocentesis at 16 weeks and 5 days of gestation. A total of 30 mL of amniotic fluid was collected under sterile conditions and divided for parallel cytogenetic and molecular analyses.

A 20 mL aliquot of amniotic fluid was cultured and harvested using standard cytogenetic protocols. Chromosome preparation was followed by G-banding using trypsin and Giemsa staining. Karyotyping was performed at a resolution of 400–550 bands. This analysis aimed to screen for chromosomal abnormalities—including common aneuploidies and large structural rearrangements such as balanced translocations and deletions/duplications (>5–10 Mb)—and to confirm fetal sex (46,XX or 46,XY), a determination critical for assessing the inheritance risk of X-linked disorders within this family.

In parallel, to ensure the reliability of subsequent genotyping, we performed a maternal cell contamination (MCC) test. We conducted short tandem repeat (STR) profiling by PCR amplification of a panel of highly polymorphic markers and compared the fetal allele pattern with that of maternal peripheral blood DNA. After confirming the absence of MCC, we then performed PCR amplification of the target genomic region using the specific primers previously validated for co-segregation analysis in the family. The amplification products were purified and subjected to Sanger sequencing on an ABI 3500 Dx DNA Analyzer. The resulting sequences were aligned with the reference sequence to determine whether the fetus carried the candidate pathogenic variant.

Additionally, an aliquot of the extracted DNA was processed using the CytoScan 750K Array (Thermo Fisher Scientific) in accordance with the manufacturer’s standard protocols. This high-resolution platform offers the ability to detect CNV as small as 50–100 kb across the genome, enabling the exclusion of clinically significant microdeletions or microduplications.

## 3 Results

### 3.1 Clinical characteristics

Among these five individuals who underwent genetic testing, three (60%) presented with unilateral renal agenesis, three (60%) displayed synkinesis, and all five (100%) had anosmia.

### 3.2 WGS

WGS of the male proband was conducted to identify potential disease-causing variants. Genome-wide analysis detected no pathogenic or likely pathogenic CNVs that could account for the observed clinical phenotype. Subsequent variant filtering and prioritization identified a rare hemizygous missense variant within the ANOS1 (NM_000216.4: c.1879T>C, p. Ser627Pro). Given that ANOS1 is a known causative gene for XLR hypogonadotropic hypogonadism 1, this variant was identified as the leading candidate. While the variant showed strong correlation with the proband’s phenotype, its classification under ACMG criteria remained a VUS at that time supported solely by the PM2_Supporting criterion (extremely low frequency in control populations). This ambiguous classification underscores the need for additional evidence, such as segregation analysis within the pedigree, to reclassify its clinical significance.

### 3.3 Sanger sequencing

**FIGURE 2:**
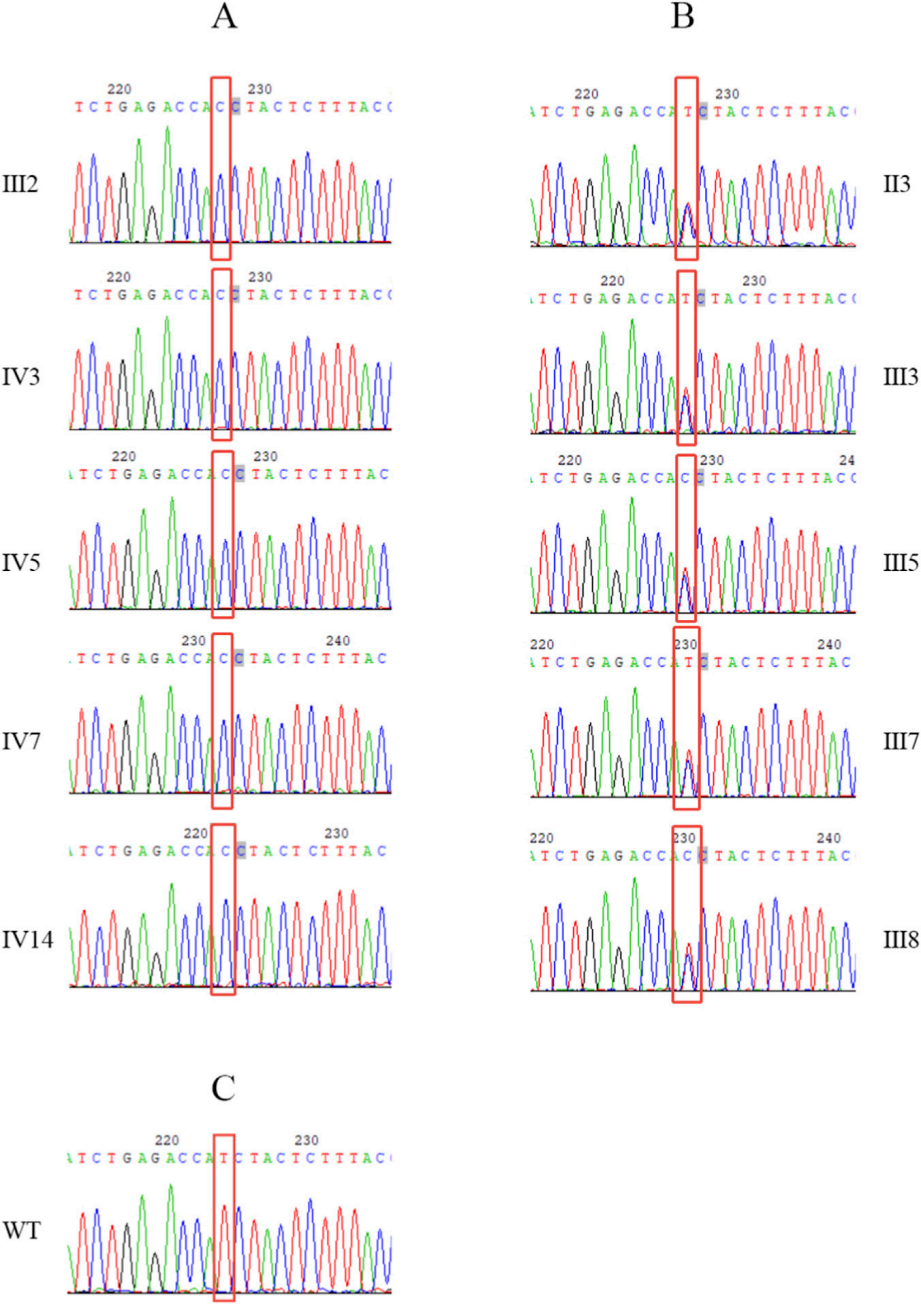
Validation of the c.1879T>C variant in ANOS1 by Sanger sequencing in 22 individuals. **(A)** Among the males, five with KS symptoms (III2, IV3, IV5, IV7, and IV14) were hemizygous for the variant. **(B)** Among the females, five (II3, III3, III5, III7, and III8) were identified as heterozygous carriers. **(C)** Wild-type control (II4).

Genotype analysis of 22 family members (10 males and 12 females) confirmed perfect co-segregation of the ANOS1 c.1879T>C variant. Among the ten males, five individuals with classic KS symptoms (III2, IV3, IV5, IV7, and IV14) were hemizygous for the variant (Figure 2A), whereas the five asymptomatic males (II4, IV4, IV6, IV9, IV10) were wild-type. Among the 12 females, five (II3, III3, III5, III7, and III8) were identified as heterozygous carriers (Figure 2B). Individual II4 served as a wild-type control (Figure 2C). As expected for XLR disorders, all heterozygous females are clinically unaffected, exhibiting normal pubertal development.

Critically, every mother of an affected male was confirmed as a heterozygous carrier: II3 (mother of III2), III3 (mother of IV3), III5 (mother of IV5 and IV7), and III7 (mother of IV14). This observed obligate carrier status across multiple generations further substantiates the variant’s pathogenicity and confirms its X-linked transmission.

**FIGURE 3:**
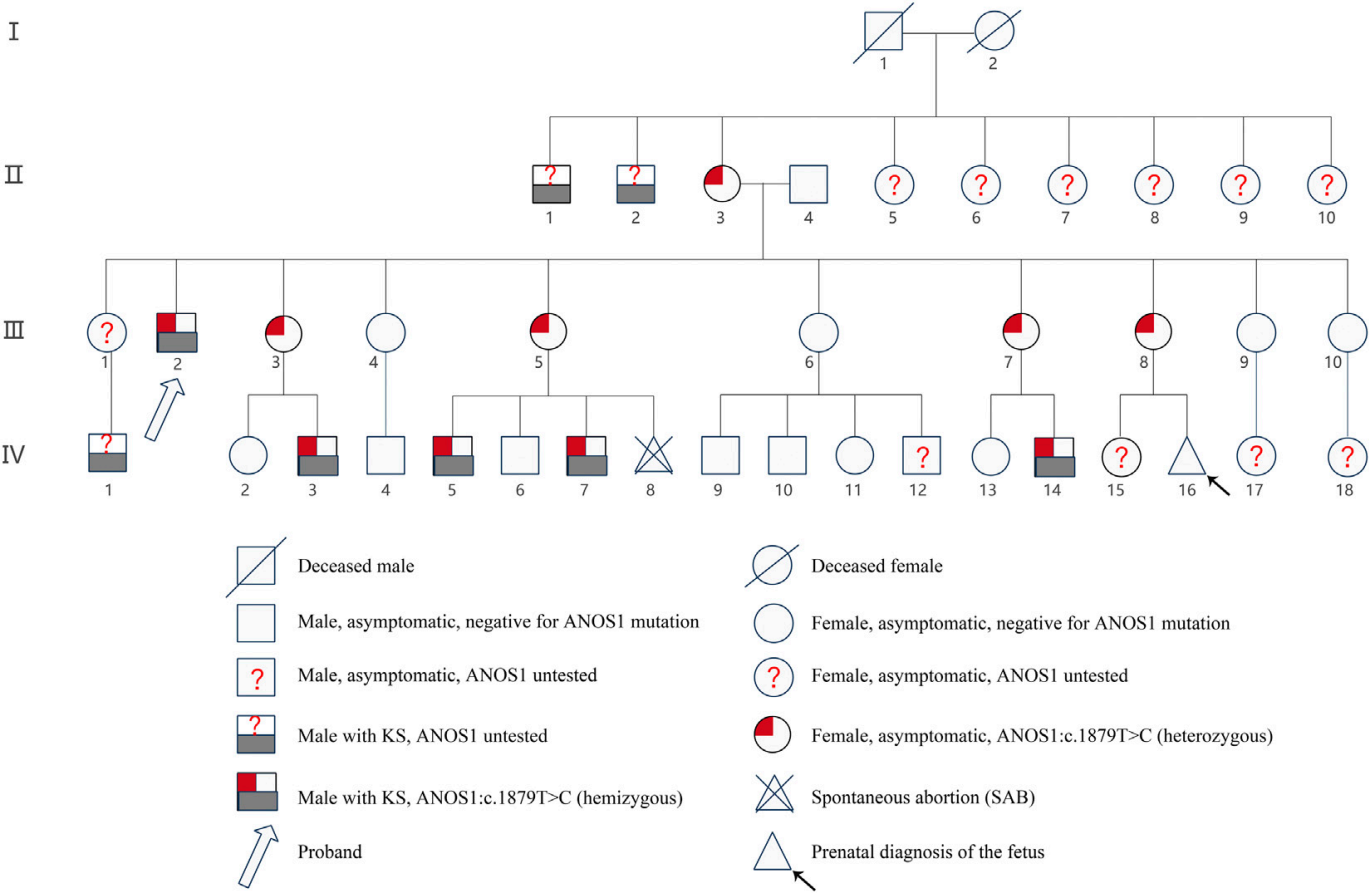
Co-segregation analysis of the c.1879T>C variant in ANOS1 with the KS phenotype in the family. Pedigree of the family shows the segregation of the c.1879T>C variant. Squares indicate males, circles indicate females, and filled black symbols represent affected individuals with KS symptoms. The proband is indicated by a black arrow. The symbols used in the pedigree and their meanings are provided below the pedigree diagram.

In summary, the ANOS1 c.1879T>C variant demonstrated complete co-segregation with the KS phenotype—present in all affected males, absent in all unaffected males, and carried by all obligate female carriers. This segregation pattern provides strong genetic evidence supporting its pathogenicity. A comprehensive summary of all genotyping results and phenotypic correlations is presented in Figure 3 and Table 2.

**TABLE 2:** Summary of ANOS1 variant segregation and phenotypic data.

No	Family ID	Sex	Age (Years)	Symptoms of KS	ANOS1: NM_000216.4: c.1879T>C
1	II3	F	74	N	Het
2	II4	M	77	N	WT
3	III2	M	49	Y	Hemi
4	III3	F	45	N	Het
5	III4	F	44	N	WT
6	III5	F	40	N	Het
7	III6	F	38	N	WT
8	III7	F	36	N	Het
9	III8	F	34	N	Het
10	III9	F	32	N	WT
11	III10	F	29	N	WT
12	IV2	F	24	N	WT
13	IV3	M	14	Y	Hemi
14	IV4	M	10	N	WT
15	IV5	M	16	Y	Hemi
16	IV6	M	14	N	WT
17	IV7	M	13	Y	Hemi
18	IV9	M	16	N	WT
19	IV10	M	13	N	WT
20	IV11	F	7	N	WT
21	IV13	F	14	N	WT
22	IV14	M	12	Y	Hemi

Abbreviations: F, female; M, male; Y, yes (presence of symptoms); N, no (absence of symptoms); WT, wild-type; Het, heterozygote; Hemi, hemizygote.

### 3.4 3D modeling of protein structure

**FIGURE 4:**
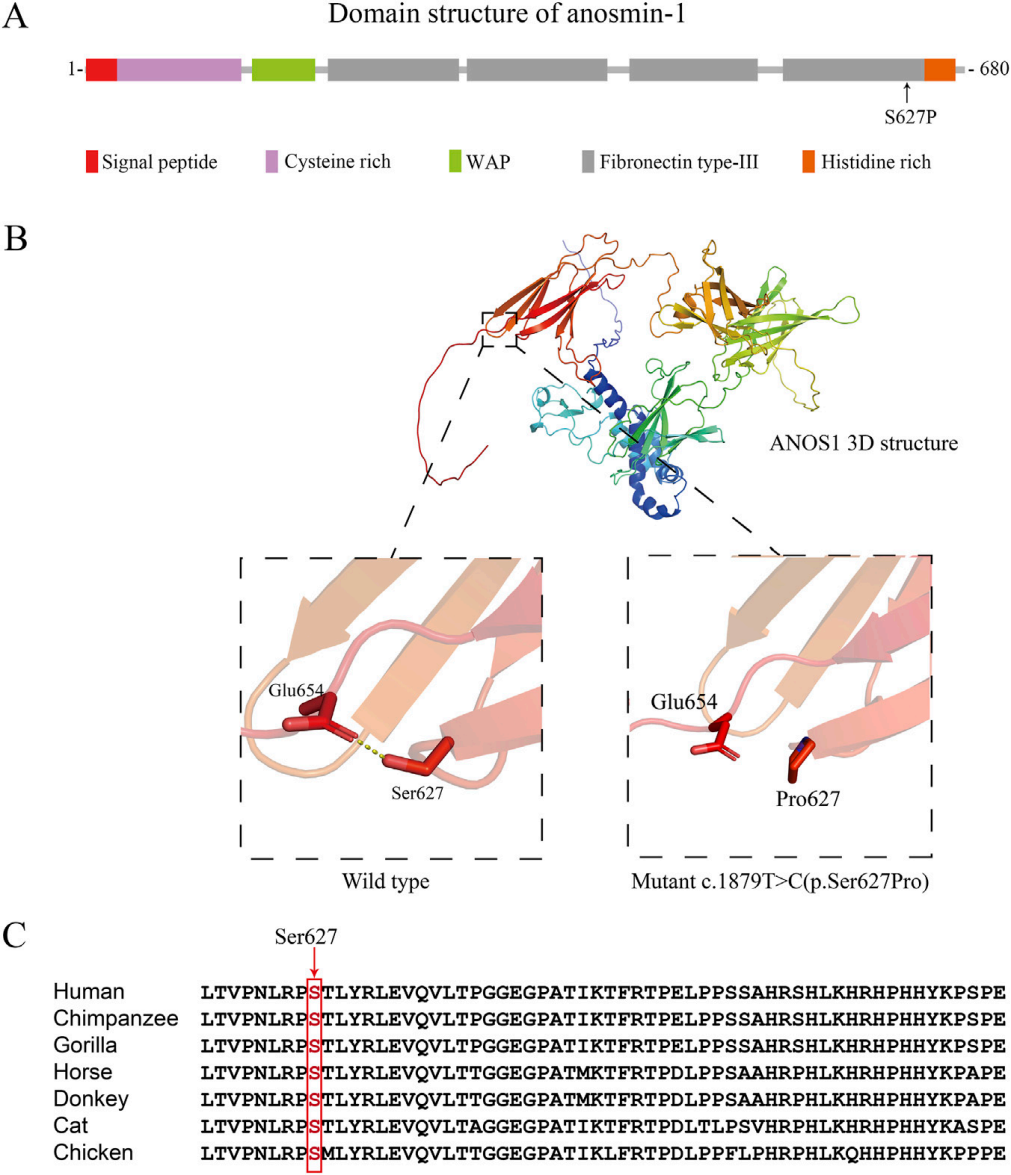
Structural and functional impact of the ANOS1 p. Ser627Pro variant. **(A)** Location of the p. Ser627Pro variant within the fourth fibronectin type III (FNIII) domain of the Anosmin-1 protein (schematic representation). **(B)** Predicted structural alteration caused by the variant. In the wild-type protein (left), the Ser627 side chain forms a hydrogen bond with Glu654 (yellow dashed line). Substitution with Pro627 (right) disrupts this interaction due to proline’s distinct structural properties and the hydrogen bond is no longer present. **(C)** Evolutionary conservation of Ser627. Sequence alignment of ANOS1 orthologs from multiple species shows that Ser627 (indicated by red box) is highly conserved.

Figure 4A shows the location of the p. Ser627Pro variant within the fourth fibronectin type III (FNIII) domain of the Anosmin-1 protein.

To assess the reliability of the predicted hydrogen-bond interactions, we calculated the average pLDDT scores for the regions surrounding residues 627 and 654. The scores were 88.4 (residues 617–637) and 69.0 (residues 644–664), indicating high and moderate confidence, respectively.

In the wildtype protein, the side chain of Ser627 in ANOS1 forms a stable hydrogen bond interaction with the side chain of the adjacent Glu654, which plays an important role in maintaining the local structural stability of the protein. However, when this site is mutated to Pro627, the original hydrogen bond network was disrupted due to the distinct structure and properties of proline (Figure 4B). This suggests that this variant may affect the structural stability of the protein, possibly increasing its susceptibility to degradation or aggregation and thereby impairing its normal function.

Further evolutionary conservation analysis reveals that Ser627 is conserved across multiple species in the sequence alignment of ANOS1 proteins. This high degree of conservation further supports the critical role of Ser627 in maintaining protein structural stability and function (Figure 4C).

### 3.5 Prenatal diagnosis

**FIGURE 5:**
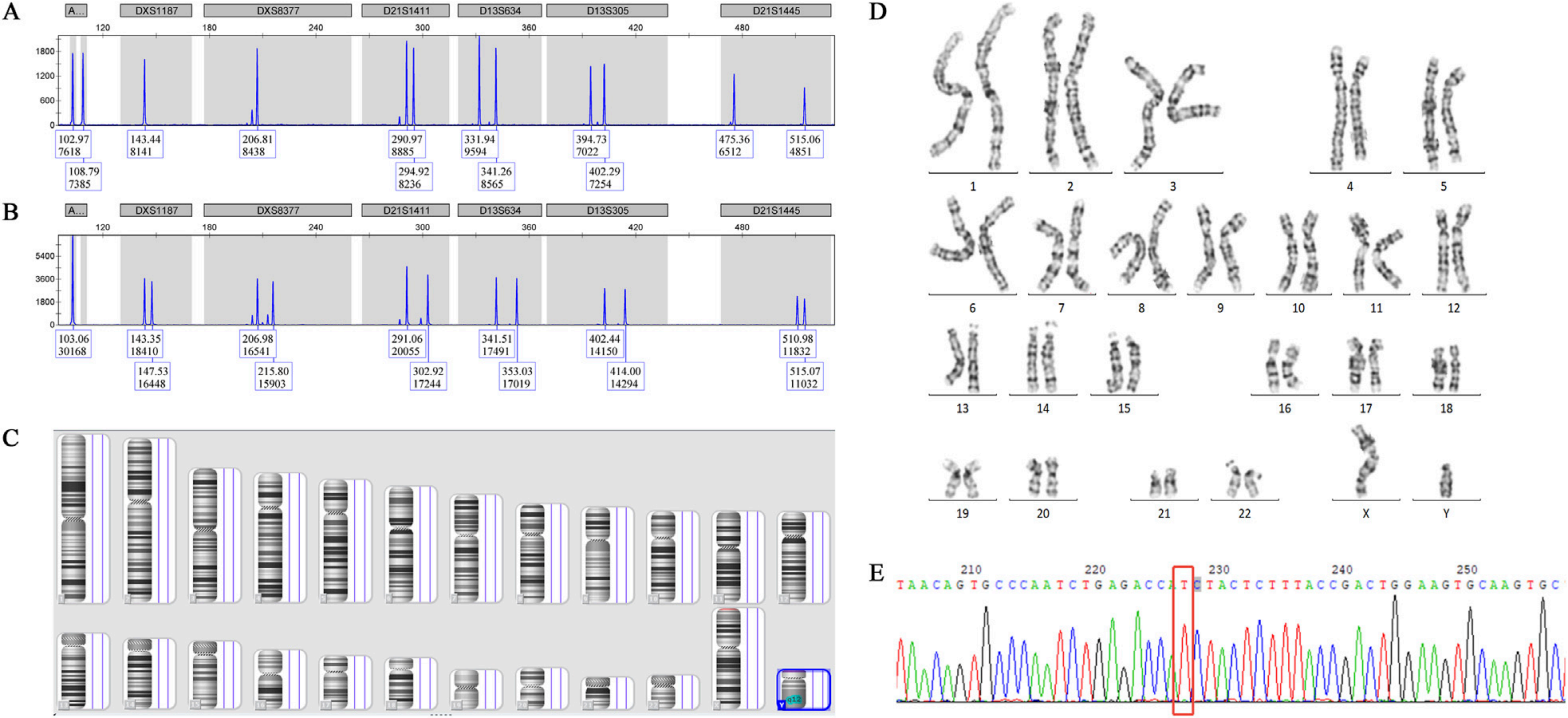
Prenatal diagnosis for the fetus (IV16) at risk of inheriting the ANOS1 c.1879T>C variant from the carrier mother (III8). **(A)** STR analysis of the fetus, and **(B)** STR analysis of the mother, confirming no MCC in the fetal sample. **(C)** Karyotyping of amniotic fluid cells revealed no abnormalities. **(D)** CMA of amniotic fluid cells revealed no abnormalities. **(E)** Sanger sequencing of amniotic fluid cells showed a wild-type genotype at the ANOS1 c.1879T>C locus, indicating that the fetus did not inherit the familial variant.

To ensure the accuracy and reliability of the prenatal diagnostic results, maternal and fetal sample identities were confirmed by short tandem repeat (STR) analysis. STR profiling was performed on both maternal blood and fetal amniotic fluid samples. The results demonstrated that the fetus shared exactly one maternal allele at all tested loci (verifying the relationship), while the absence of extra peaks ruled out MCC. This validation step was critical to ensure that all subsequent genetic analyses accurately reflected the fetal genotype (Figures 5A,B).

Conventional karyotyping of cultured amniotic fluid cells revealed a normal male karyotype (46,XY), with no evidence of numerical or structural chromosomal abnormalities (Figure 5C). In parallel, chromosomal microarray analysis (CMA) was performed on uncultured amniotic fluid cells to detect submicroscopic CNV. The CMA results were also normal, indicating the absence of pathogenic microdeletions/microduplications, or regions of loss of heterozygosity that could contribute to clinical abnormalities (Figure 5D).

Considering the XLR pattern of the condition in this family, the 46,XY karyotype indicates a 50% risk for the fetus of having inherited the likely pathogenic variant. Given the family’s known history of this variant in the ANOS1 (c.1879T>C, p. Ser627Pro), targeted Sanger sequencing was conducted on DNA extracted from amniotic fluid cells. The sequencing results showed a wild-type genotype at the c.1879 locus, with only the thymine (T) base detected and no evidence of the cytosine (C) variant. This finding confirmed that the fetus did not inherit the familial ANOS1 variant (Figure 5E).

No structural abnormalities were detected on prenatal ultrasound. Following comprehensive genetic counseling, the pregnant woman elected to proceed with the pregnancy, and long-term clinical follow-up is ongoing to assess the infant’s development.

## 4 Discussion

In this study, we report a novel missense variant (p.Ser627Pro) in the ANOS1 identified in a large Chinese family with KS. Through comprehensive segregation analysis across family members, we demonstrate complete co-segregation of this variant with the disease phenotype, providing strong genetic evidence for its pathogenicity. We also successfully performed prenatal diagnosis for a male fetus with 50% risk based on this finding. These findings collectively expand the mutational spectrum of ANOS1 and demonstrate the clinical utility of family-based investigation and reproductive genetic testing for KS.

The incidence of CHH is approximately 1–10 per 100,000 newborns, with a significant gender disparity. For KS, a Finnish study reported incidences of ∼1:30,000 in males and ∼1:125,000 in females (Laitinen et al., 2011). KS pathogenesis is closely linked to GnRH neuron development and migration. GnRH neurons, olfactory neurons, and olfactory ensheathing cells share a common origin in the nasal placode. During embryogenesis, GnRH neurons migrate from the vomeronasal organ along the vomeronasal nerve axons to reach the forebrain (Kumar Yadav et al., 2025). Disruption at any stage of this process can lead to impaired GnRH secretion and consequent disease manifestation. Clinically, KS is diagnosed in adolescents with absent or partial puberty based on biochemical evidence of hypogonadism caused by impaired GnRH-mediated LH and FSH release (Sonne et al., 2026). Over 50 genes are implicated in CHH, involved in GnRH neuronal fate specification, migratory guidance, synaptic integration, and GnRH synthesis/secretion, contributing to the high genetic heterogeneity of this disorder (Fausto et al., 2026). This clinical and genetic complexity poses significant challenges to the precise genetic diagnosis of KS.

To address these challenges, we established and demonstrated an integrated research strategy that combined systematic familial investigation and multidimensional clinical phenotyping, supplemented by AlphaFold3. This approach is intended to provide an effective methodological pathway for overcoming the diagnostic difficulties associated with such complex genetic disorders.

ANOS1, located on Xp22.31, composed of 14 exons and encodes a 680-amino acid extracellular adhesion protein named anosmin-1. This protein contains an N-terminal cysteine-enriched Cys-box domain, a whey acidic protein (WAP) domain, 4 fibronectin type III (FnIII) domains, and a histidine-rich C-terminal region (Nie et al., 2017). The c.1879T>C variant in ANOS1 is located in exon 13 and is absent in population databases (PM2_Supporting, +1 point) without prior reports in ClinVar, HGMD, or PubMed. This change causes a p. Ser627Pro substitution, which replaces a polar serine with a nonpolar proline and is predicted to disrupt a stabilizing hydrogen bond between Ser627 and Glu654, likely impairing protein stability and function. Therefore, based solely on population frequency and *in silico* prediction data, the variant would be classified as a VUS. Furthermore, the variant fully co-segregates with the XLR phenotype in this family. The proband’s father did not carry the variant, and the proband inherited the variant from his heterozygous mother. Excluding the proband from the co-segregation analysis to avoid ascertainment bias, we identified four affected males carrying the variant and four unaffected males without the variant, providing a total of eight informative meioses. According to the ClinGen co-segregation scoring framework, each informative meiosis contributes +1 point. Thus, the total calculated evidence would be +8 points, which exceeds the maximum cap of +5.0 points for evidence (PP1 and PP4). We therefore applied the capped score of +5.0 points, which designates the evidence level as PP1_Strong and adds +5 points toward the pathogenic classification (Tavtigian et al., 2018; Biesecker et al., 2024). Based on this combined evidence, the variant is classified as likely pathogenic. Per ACMG/AMP variant interpretation guidelines, the terms “pathogenic variant” and “likely pathogenic variant” are synonymous in a clinical setting, meaning that both are considered diagnostic and can be used for clinical decision making (Richards et al., 2015).

The p. Ser627Pro variant is located in the fourth FNIII domain of the Anosmin-1 protein. The substitution of serine with proline at this position is predicted to disrupt critical local hydrogen-bonding interactions, particularly with Glu654, leading to impaired domain stability and altered conformation. These structural perturbations likely compromise the protein’s function as an extracellular matrix signaling molecule. To date, approximately forty missense variants have been reported to cause KS, distributed across various domains of Anosmin-1. Among these, mutations including Val560Ile, Gly567Ser, His568Gln, Trp571Arg, and Gln635Pro are also localized to the fourth FNIII domain (Zhang et al., 2013; Mao et al., 2015; Takagi et al., 2014; Albuisson et al., 2005; Zeng et al., 2022). However, as noted in the literature, there is also no correlation between phenotype and location of the mutation. Different mutations within the same domain—or even at the same residue—can give rise to variable associated phenotypic profiles across different families (Gonçalves et al., 2017). This strongly suggests that clinical manifestations are determined by a complex system shaped by the spatiotemporal expression patterns and functional pleiotropy of Anosmin-1 across multiple developmental tissues. The incomplete penetrance of unilateral renal agenesis likely reflects the influence of other kidney development-related genetic pathways on ANOS1 function (Tickotsky and Moskovitz, 2014; Stamou et al., 2019). A further layer of phenotypic heterogeneity is reflected in the neurological manifestations of KS: the spectrum of cerebral morphological changes identified in KS individuals with mirror movements may constitute the structural correlates of the aberrant reorganization of complex networks supporting fine manual dexterity (Renukanthan et al., 2015).

In this study with five affected males all harboring the p. Ser627Pro variant, the two defining core phenotypes—olfactory dysfunction and CHH—were fully penetrant (100%). Notably, bilateral cryptorchidism also demonstrated complete penetrance in this pedigree. This pattern aligns closely with literature reports stating that “almost all ANOS1 mutation patients exhibit olfactory abnormalities” and that “ANOS1 mutations have high penetrance,” underscoring the pivotal role of this gene in olfactory axon guidance and GnRH neuron migration (Zeng et al., 2022). Beyond these three fully penetrant phenotypes, significant clinical heterogeneity was observed: synkinesis was present in three, and three members had unilateral renal agenesis. This pattern of “constant core phenotypes with variable associated phenotypes” is a hallmark characteristic of ANOS1-related disorders.

In summary, this study confirms p. Ser627Pro as a novel likely pathogenic variant in ANOS1 through comprehensive phenotypic and co-segregation analyses in a familial cohort, thereby expanding the mutational spectrum of this gene. Furthermore, by integrating our findings with the existing literature, we elucidate the phenomenon of “variable expressivity from an identical mutation” in monogenic disorders. This insight carries crucial clinical implications: for individuals diagnosed with or carrying ANOS1 variants, clinical management must extend beyond the core endocrine and olfactory manifestations to implement lifelong multidisciplinary surveillance. Routine evaluations are warranted, including renal ultrasonography for congenital anomalies, neurological assessment for synkinesis, and detailed examination of the male reproductive system. Such a proactive, integrated approach is fundamental to achieving truly individualized precision medicine in KS, enabling early intervention, preserving fertility, and improving long-term quality of life. Beyond the management of affected individuals, the identification of likely pathogenic ANOS1 variants has direct implications for reproductive genetics. KS can be diagnosed on the basis of clinical symptoms and family history, supported by biochemical testing and definitively confirmed by genetic analysis. In this context, prenatal diagnosis and preimplantation genetic testing represent important measures for reducing birth defects and preventing the intergenerational transmission of genetic disorders.

## 5 Limitations of the study

This study has several limitations. First, functional experiments were not performed to experimentally validate the impact of the p. Ser627Pro variant on anosmin-1 protein. As with all computational predictions, the AlphaFold3 provides a static snapshot and cannot capture dynamic cellular environments or confirm the functional consequence of the predicted hydrogen bond disruption. However, this does not affect the variant classification, as the likely pathogenic designation is supported by genetic evidence (PP1_Strong, +5 points; PM2_Supporting, +1 point), which independently reaches the threshold for likely pathogenic. The structural prediction presented in this study is intended to provide a mechanistic hypothesis rather than serve as an independent line of evidence for classification. Second, the molecular basis for the observed phenotypic heterogeneity within this family remains to be elucidated. Addressing these limitations in future work will deepen our understanding of ANOS1-related KS and optimize patient care.

## Data Availability

The original contributions presented in the study are publicly available. This data can be found in the China National Center for Bioinformation, GSA for Human repository with the accession number HRA020003, at https://ngdc.cncb.ac.cn/gsa-human/browse/HRA020003.
